# Characterization of pendrin in urinary extracellular vesicles in a rat model of aldosterone excess and in human primary aldosteronism

**DOI:** 10.1038/s41440-021-00710-5

**Published:** 2021-07-29

**Authors:** Fumika Ochiai-Homma, Emiko Kuribayashi-Okuma, Yuya Tsurutani, Kenichi Ishizawa, Wataru Fujii, Kohei Odajima, Mika Kawagoe, Yoshihiro Tomomitsu, Masataka Murakawa, Shinichiro Asakawa, Daigoro Hirohama, Michito Nagura, Shigeyuki Arai, Osamu Yamazaki, Yoshifuru Tamura, Yoshihide Fujigaki, Tetsuo Nishikawa, Shigeru Shibata

**Affiliations:** 1 Division of Nephrology, Department of Internal Medicine, Teikyo University School of Medicine, Tokyo, Japan; 2 Endocrinology and Diabetes Center, Yokohama Rosai Hospital, Yokohama, Japan

**Keywords:** Mineralocorticoid, Transporter, Urine, Exosome

## Abstract

Pendrin is a Cl^−^/HCO_3_^−^ exchanger selectively present in the intercalated cells of the kidney. Although experimental studies have demonstrated that pendrin regulates blood pressure downstream of the renin-angiotensin-aldosterone system, its role in human hypertension remains unclear. Here, we analyzed the quantitative changes in pendrin in urinary extracellular vesicles (uEVs) isolated from a total of 30 patients with primary aldosteronism (PA) and from a rat model of aldosterone excess. Western blot analysis revealed that pendrin is present in dimeric and monomeric forms in uEVs in humans and rats. In a rodent model that received continuous infusion of aldosterone with or without concomitant administration of the selective mineralocorticoid receptor (MR) antagonist esaxerenone, pendrin levels in uEVs, as well as those of epithelial Na^+^ channel (ENaC) and Na-Cl-cotransporter (NCC), were highly correlated with renal abundance. In patients with PA, pendrin levels in uEVs were reduced by 49% from baseline by adrenalectomy or pharmacological MR blockade. Correlation analysis revealed that the magnitude of pendrin reduction after treatment significantly correlated with the baseline aldosterone-renin ratio (ARR). Finally, a cross-sectional analysis of patients with PA confirmed a significant correlation between the ARR and pendrin levels in uEVs. These data are consistent with experimental studies showing the role of pendrin in aldosterone excess and suggest that pendrin abundance is attenuated by therapeutic interventions in human PA. Our study also indicates that pendrin analysis in uEVs, along with other proteins, can be useful to understand the pathophysiology of hypertensive disorders.

## Introduction

Primary aldosteronism (PA) is a common form of secondary hypertension [1, 2] and has been shown to be associated with increased risks of cardiovascular and kidney diseases [3–5]. Excessive production of the steroid hormone aldosterone results in increased activity of NaCl-transporting proteins in renal tubules, thereby increasing fluid volume and blood pressure (BP). Experimental evidence indicates that apical membrane channels and transporters such as the epithelial Na^+^ channel (ENaC), Na-Cl-cotransporter (NCC), and Cl^−^/HCO_3_^−^ exchanger pendrin are dysregulated under aldosterone excess [6–8]. However, few studies have examined whether the reported mechanisms in animal models apply to PA in humans.

Pendrin, encoded by *SLC26A4*, is selectively present in β-intercalated cells and non-α, non-β intercalated cells of the kidney, mediating Cl^−^ reabsorption in exchange for HCO_3_^−^ secretion [7]. Experimental studies using animal models have demonstrated the importance of pendrin in regulating BP. Mice lacking pendrin have been shown to have reduced BP in response to NaCl restriction [9, 10] and fail to show a pressor response to mineralocorticoids [11]. Conversely, transgenic mice that overexpress pendrin showed salt-dependent hypertension [12]. Moreover, double-knockout mice lacking pendrin and NCC showed severe salt wasting and volume depletion, confirming the compensatory roles of these proteins in fluid volume homeostasis [13]. As an upstream signaling component, pendrin is regulated by multiple mechanisms, including the renin-angiotensin-aldosterone (RAA) system, acid/base changes, and potassium and chloride balance [14–20]. In volume depletion, pendrin is stimulated by angiotensin II (AngII) and MR [9, 21, 22], and we have reported the signaling interaction between AngII and MR, in which AngII dephosphorylates MR at S843 through a mechanism involving mammalian target of rapamycin and unc51-like kinase 1, promoting MR signaling and pendrin induction [10, 23, 24]. Regarding the role of pendrin in BP regulation in humans, one study investigated BP levels in subjects with biallelic *SLC26A4* mutations. In this study, the authors found that both systolic and diastolic BP were lowered by 4–6 mmHg in these patients compared with age-matched controls [25]. Nonetheless, the evidence regarding the role of pendrin in BP regulation in humans is still limited.

In eukaryotic cells, nanosized extracellular vesicles (EVs), including exosomes, are released from the multivesicular body into extracellular fluids, including plasma and urine [26, 27]. These particles carry proteins that are present in their cells of origin, suggesting that they may potentially serve as biomarkers of disease risk and progression. In human urine, exosomes have been successfully identified by differential centrifugation [28, 29], and several studies have investigated their biological importance in healthy and disease states [30, 31]. A large-scale proteomic study of urinary EVs in healthy individuals detected many salt and water transporters, including pendrin [32]. Another proteomic study analyzed acute changes in response to a high- or low-salt diet in essential hypertension and reported that pendrin peptides were increased by salt restriction [33], a finding that is consistent with observations in animal models. Alterations in pendrin abundance in urinary EVs in response to NaCl, NH_4_Cl, and NaHCO_3_ loading have also been reported in healthy individuals [34]. Based on these observations, we aimed to determine the changes in pendrin levels in EVs isolated from the urine of patients with PA at baseline and during treatment. Using an animal model of PA, we also tested the biological importance of pendrin in urinary EVs.

## Methods

### Patients and study design

This observational study included a total of 30 patients who were diagnosed with PA in Yokohama Rosai Hospital or in Teikyo University Hospital. Cushing syndrome was excluded in all cases by the baseline adrenocorticotropic hormone (ACTH) and cortisol levels and by the dexamethasone suppression test. The study protocol was approved by the institutional review board at each institution, and all patients provided written informed consent. The study was conducted according to the principles expressed in the Declaration of Helsinki. In the first set of studies, spot urine was collected for the isolation of urinary EVs both at baseline (without MR antagonist treatment) and at the follow-up visit (at least 3 months after pharmacological or surgical treatment) in 13 patients with PA. The changes in pendrin levels in urinary EVs were compared before and after the treatment for each of the participants. In the second set of studies, spot urine was collected from 28 patients with PA who were not on MR antagonists (among whom 11 patients also participated in the first set of studies), and the association of pendrin levels in urinary EVs with biochemical parameters was analyzed.

### Isolation of EV-enriched fractions from urine

Isolation of human urinary EVs was performed in accordance with previous reports [29]. An aliquot (50 ml) of human urine samples was immediately placed in a sterile container supplemented with protease inhibitor cocktail (Roche Diagnostics, Switzerland). After centrifugation at 1500 × *g* for 10 min at 4 °C to remove insoluble materials, urine samples were centrifuged at 17,000 × *g* for 15 min. The supernatant was saved, and the 17,000 × *g* pellets were resuspended in an isolation solution (10 mM triethanolamine, 250 mM sucrose, pH 7.6), followed by incubation with DTT (final concentration: 200 mg/ml) to disrupt the polymeric network [29]. The samples were then centrifuged again at 17,000 × *g* for 10 min. The two supernatants from the 17,000 × *g* spins were pooled together and were further centrifuged at 200,000 × *g* for 60 min.

### Animal experimental procedures

Animal procedures were approved by the Teikyo University Ethics Committee for Animal Experiments (#20-007) and were conducted in accordance with the guidelines of Teikyo University. Male Sprague-Dawley rats at six weeks of age were obtained from Tokyo Laboratory Animals Science (Japan). Rats received continuous infusion of aldosterone at a dose of 0.75 μg/h via a minipump (Alzet, USA) after uninephrectomy (Aldo group). The dose of aldosterone was in accordance with our previous experiments [17, 35]. A subgroup of aldosterone-infused rats received esaxerenone, a nonsteroidal MR antagonist (mixed in chow, 0.001%; corresponding to 1 mg/kg body weight/day; Aldo+Esax). Control rats underwent a sham operation (we confirmed that the pendrin levels were similar between the sham-operated rats and the uninephrectomized rats receiving continuous infusion of vehicle; data not shown). All rats received standard chow containing 0.3% NaCl (CRF-1; Oriental Yeast, Japan) throughout the experiment. Esaxerenone was provided by Daiichi Sankyo Co., Ltd (Japan).

At four weeks, urine was collected for 24 h by using individual metabolic cages (Natsume KN-646, Japan) and a container supplemented with protease inhibitor. The container was cooled on blue ice to avoid protein degradation during urine collection. Collected urine samples were immediately centrifuged and processed for the isolation of urinary EVs as described above. Urinary albumin and electrolyte levels were measured by ELISAs (SRL, Japan). Urinary electrolyte concentrations were measured by ion-selective electrode methods. Systolic BP was measured using volumetric pressure recording (CODA noninvasive blood pressure system; Kent Scientific, USA) [36]. This method has been validated to provide accurate BP measurements and is highly correlated with the telemetry method in rats [37]. To minimize the influence of diurnal variation, we measured BP at approximately the same time in the afternoon. For each rat, we calculated the mean ± SEM of ≥10 recordings. Animals were then euthanized under anesthesia of inhaled isoflurane. Blood samples were obtained by vena cava puncture. Kidneys were removed, snap-frozen, and stored at −80 °C until use. Serum electrolyte levels were measured by an iSTAT blood gas analyzer (Abbott, USA).

### Electron microscopy of urinary EVs

Pellets obtained from the 200,000 × *g* centrifugation as described above were resuspended in phosphate-buffered saline, and the suspension was applied to carbon-coated grids. The samples were then stained with 2% uranyl acetate and examined using an electron microscope (H-7600, Hitachi, Japan) [38].

### Western blotting

For urinary EVs, the pellets were resuspended in isolation solution and added to Laemmli sample buffer. Sample loading was normalized to urinary creatinine concentration. Samples were separated on polyacrylamide gels and transferred to PVDF membranes [36]. The membrane was then incubated with anti-pendrin antibody (kindly provided by Peter Aronson at Yale University), followed by peroxidase-conjugated anti-rabbit antibodies (GE, USA). Signals were visualized by imaging using ECL reagents (Western Blot Hyper, TaKaRa, Japan). The pendrin antibody used in this study has extensively been characterized in previous studies [10, 14, 23, 39] and was further validated in the current study. The membrane was then stripped and reprobed with an antibody against Alix (Proteintech, USA), an exosomal marker, to confirm the isolation of exosomes and for normalization. The Alix signal was visualized using ECL reagents from Perkin Elmer (USA). Other antibodies included anti-NCC (Millipore) and anti-ENaCγ (StressMarq) [17]. For evaluation of pendrin abundance across multiple Western blots in a cross-sectional study, common standard samples (rat kidney lysates) were included in each blot for normalization.

In rat kidneys, pendrin abundance was analyzed in the plasma membrane-enriched fraction that was prepared using a plasma membrane isolation kit (Minute, Invent Biotechnologies, USA). Enrichment of plasma membrane proteins was validated in our previous studies [17, 36]. Equal amounts of protein were mixed with Laemmli sample buffer, and Western blot analysis was performed as described above. For plasma membrane proteins, CBB staining was used to ensure equal loading of different samples.

### Peptide competition assay

A peptide blocking assay was performed in accordance with a previous report [39]. An immunizing peptide for anti-pendrin antibody (CKDPLDLMEAEMNAEELDVQDEAMRRLAS; corresponding to C-terminal 29 aa. of rat pendrin) was synthesized at GenScript (USA). The peptide was then incubated with anti-pendrin antibody for 16 h at 4 °C, followed by centrifugation at 18,000 × *g* for 15 min at 4 °C. The supernatant was then used for Western blot analysis.

### Immunofluorescence study

Tissues were fixed in 4% paraformaldehyde (PFA) for 4 h at 4 °C. Tissues were then incubated in 30% sucrose in PBS overnight at 4 °C, mounted in OTC (Tissue-Tek), and frozen until use. After the samples were blocked with Protein Block (Dako, Denmark), cryosections (0.2 μm thick) were stained with the indicated primary antibodies, followed by anti-rabbit secondary antibody conjugated with Alexa Fluor 488 or anti-goat antibody conjugated with Alexa Fluor 596. The primary antibodies used included antibodies against pendrin and AQP2 (Santa Cruz Biotechnology, USA) [23].

### Statistics

The data are summarized as the mean ± SD for continuous variables and as absolute numbers and percentages for categorical values. GraphPad Prism software, version 7.05 (GraphPad Software, Inc., USA), was used for statistical analyses. An unpaired *t* test was used for comparisons between two groups. For multiple comparisons, statistical analysis was performed by ANOVA followed by Tukey post hoc tests. A paired *t* test was used to assess the changes in pendrin levels over time. Correlations between parameters were analyzed by Pearson’s correlation test. Logarithmic transformation was applied for ARR, plasma aldosterone, PRA, and urinary Na^+^/K^+^ prior to correlation analysis because these variables showed skewed distributions. All statistical tests were two-sided. A *P* value < 0.05 was considered statistically significant.

## Results

### Detection of monomeric and dimeric forms of pendrin in urinary EVs isolated from patients with PA

**Fig. 1:**
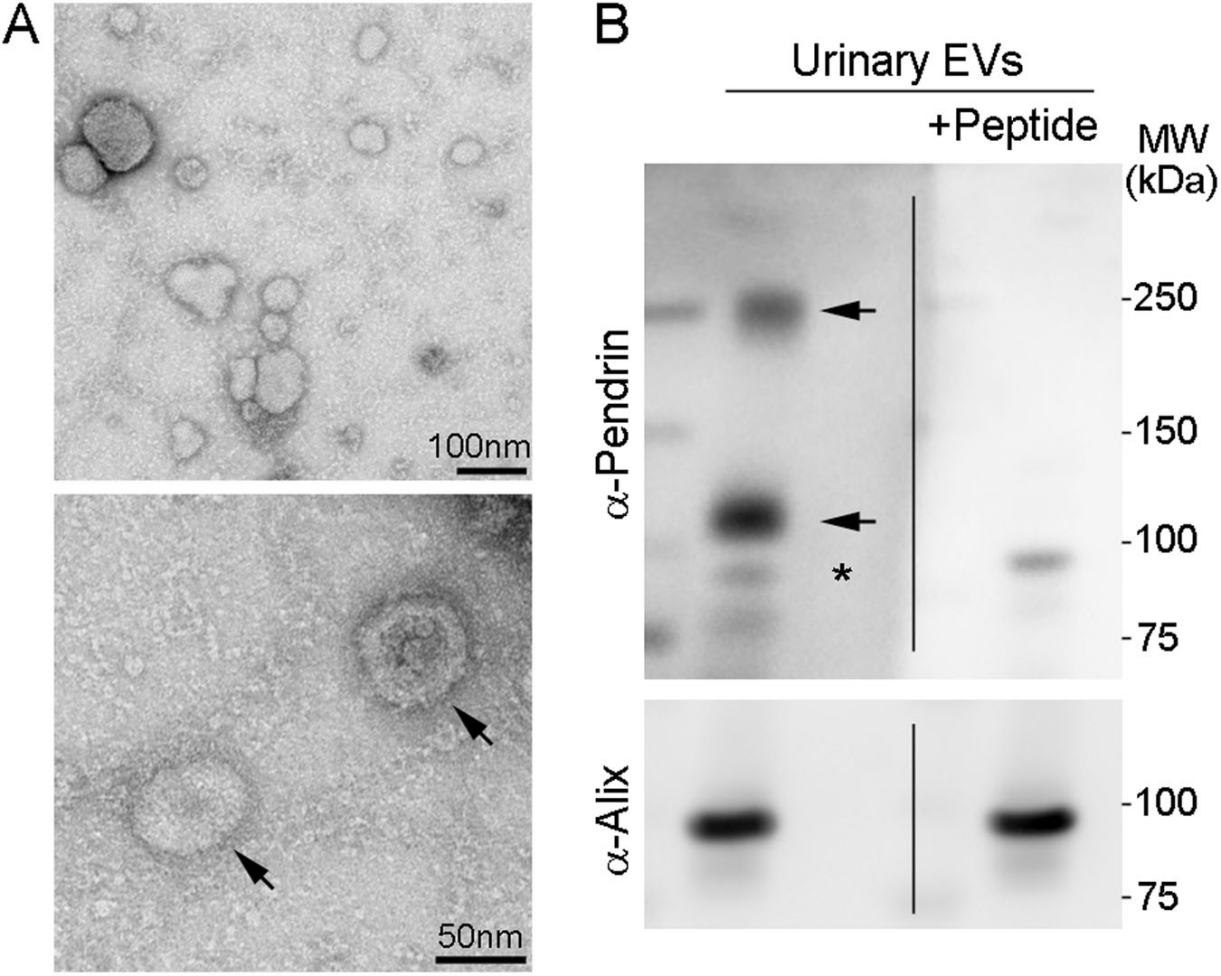
Detection of pendrin in urinary extracellular vesicles (EVs) isolated from patients with primary aldosteronism (PA). **A** Representative electron microscope images of the EV-enriched fraction. Arrows indicate urinary exosome-like particles. **B** (Top panel) A urinary EV sample isolated from a patient with PA subjected to Western blotting with anti-pendrin (top panel). Arrows indicate monomer (~120 kDa) and dimer (~240 kDa) forms of pendrin. For confirmation of the signal specificity, the same sample was analyzed by Western blotting with anti-pendrin antibody after preincubation with the immunizing pendrin peptide (right). Incubation of the antibody with the immunizing peptide completely eliminates the signals at both ~120 and ~240 kDa. An asterisk at ~90 kDa denotes nonspecific signal. The membranes were then stripped and reprobed with anti-Alix, a marker of exosomes (bottom panel).

We first characterized pendrin signals in urinary EVs isolated from patients with PA. As shown in Fig. 1A, electron microscopic analysis of the sampled confirmed the enrichment of exosome-like particles (20–100 nm in size), validating the sample quality [29]. Western blot analysis of urinary EVs using anti-pendrin antibody demonstrated two immunoreactive bands at ~120 kDa and ~240 kDa, representing monomeric and dimeric forms, respectively (Fig. 1B). Both signals were completely eliminated by competition with the immunizing pendrin peptide (Fig. 1B), confirming that pendrin exhibits dimeric organization in urinary EVs. In the following analysis, both forms were included to determine the pendrin abundance in urinary EVs.

### Correlation of pendrin levels in urinary EVs with renal pendrin abundance in a rodent model of PA

**Fig. 2:**
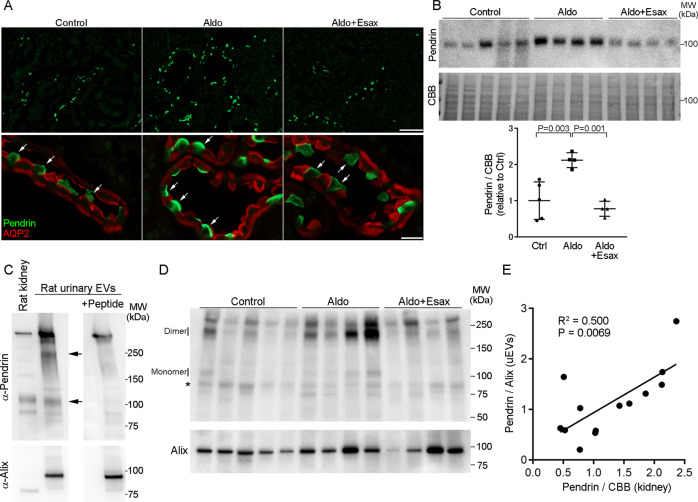
Analysis of pendrin abundance in the kidney and in urinary EVs in aldosterone-infused rats and those receiving esaxerenone, a nonsteroidal mineralocorticoid receptor (MR) antagonist. **A** Kidney sections from the control rats, aldosterone-infused rats (Aldo), and aldosterone-infused rats receiving esaxerenone (Aldo + Esax) were stained for pendrin (green) and aquaporin-2 (AQP2; red), which is a marker for the principal cells of the collecting duct. Bars indicate 100 μm (top panel) and 20 μm (bottom panel). Pendrin (arrows) levels increased at the apical membrane of AQP2-negative intercalated cells in the aldosterone-infused rats, which was blocked by esaxerenone. **B** Expression of pendrin in the plasma membrane-enriched fraction of the kidneys from the indicated rats was analyzed by Western blotting. Blots show biological replicates. Dot plot graphs show the results of quantitation. Data are expressed as the mean ± SD. **C** Rat urinary EVs were subjected to Western blotting with anti-pendrin (left panel) or anti-pendrin antibody preincubated with the immunizing peptide (right panel). Similar to human samples, both bands at ~120 kDa and ~240 kDa, representing monomeric and dimeric forms of pendrin (arrows), were eliminated by blocking with immunizing peptide. In the bottom panels, the same membranes were reprobed with anti-Alix after stripping. **D** EVs were isolated from urine obtained from the control, Aldo, and Aldo+Esax rats and subjected to Western blot analysis for pendrin. **E** Correlation between pendrin levels in the kidney and those of urinary EVs across groups.

Although urinary EVs have been postulated to be a useful biomarker of various kidney disorders [32, 40], their relevance in kidney biology is unclear [41]. We next tested the physiological importance of pendrin in urinary EVs using a rat model of aldosterone excess. Sprague-Dawley rats received continuous infusion of aldosterone after uninephrectomy. A subgroup of aldosterone-infused rats concomitantly received esaxerenone, an MR antagonist. After 4 weeks, the pendrin levels in the kidney as well as those in urinary EVs were determined. Biological parameters are shown in Supplementary Table 1. As reported previously in similar models [11], pendrin was highly detected at the apical membrane of aquaporin-2-negative intercalated cells in the aldosterone-infused rats (Fig. 2A). However, the pendrin signals in the aldosterone-infused rats receiving esaxerenone were comparable to those in the control animals (Fig. 2A). Western blot analysis confirmed that pendrin abundance in the kidney was significantly increased by aldosterone (2.1-fold increase), which was almost completely blocked by esaxerenone (Fig. 2B).

We then analyzed the association of pendrin levels in urinary EVs (corrected for Alix) with levels in the kidney (corrected for CBB density). Consistent with the results in human urine samples, pendrin in rat urinary EVs was detected at ~240 kDa for dimeric and ~120 kDa for monomeric forms (Fig. 2C). These signals were completely eliminated again by preincubation with the immunizing pendrin peptide (Fig. 2C). An additional band at ~300 kDa was not eliminated by the blocking peptide, suggesting that the band contains a nonspecific signal. We quantified pendrin abundance in urinary EVs and compared the levels with the renal pendrin levels. Correlation analysis revealed that pendrin levels in urinary EVs were highly correlated with pendrin abundance in the kidney (*R*^2^ = 0.500; *P* = 0.0069) (Fig. 2D and E), confirming that Western blot analysis of pendrin in urinary EVs can predict the levels of pendrin abundance in the kidney under aldosterone excess.

**Fig. 3:**
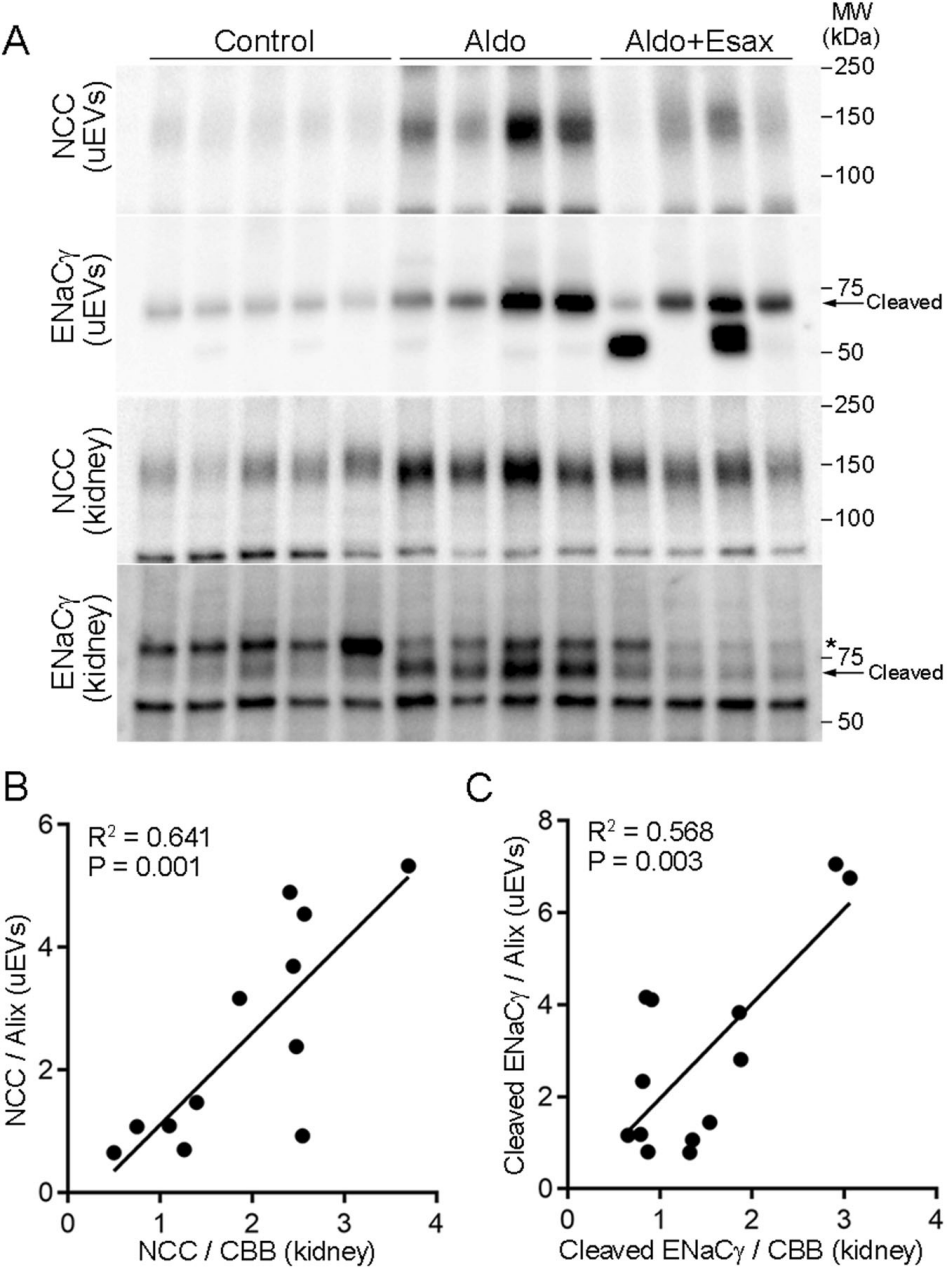
Analysis of NCC and ENaC in urinary EVs in the aldosterone-infused rats and those receiving esaxerenone. **A** Urinary EVs and plasma membrane-enriched fraction of the kidneys prepared as described in Fig. 2 were subjected to Western blot analysis using NCC and ENaCγ antibodies. Asterisk indicates uncleaved form. Correlation analysis for NCC (**B**) and ENaCγ (**C**).

Previous studies have documented acute changes in the ENaC and NCC levels in urinary EVs after mineralocorticoid administration [33, 42]. We additionally evaluated whether the ENaC and NCC levels in urinary EVs were associated with the renal levels in a rat model that received chronic aldosterone infusion with or without esaxerenone. Similar to the pendrin results, we found that both cleaved ENaCγ and NCC levels in urinary EVs were significantly correlated with those in the kidney (Fig. 3). These data are consistent with a previous study [43] and further confirm the usefulness of urinary EV analysis.

### Reduction of pendrin in urinary EVs after treatment of the patients with PA

**Fig. 4:**
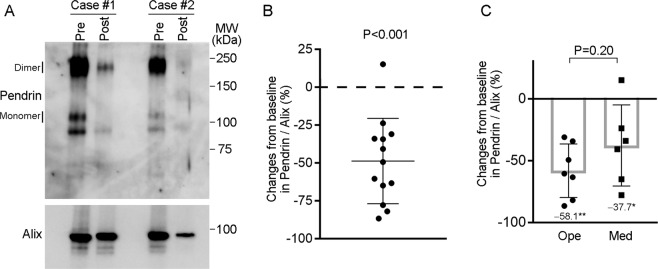
Pre- and post-treatment analysis of pendrin in urinary EVs isolated from subjects with PA. **A** Representative immunoblots of pendrin in urinary EVs isolated from subjects with PA before (pre) and after (post) treatment. **B** Changes in pendrin levels from baseline in 13 subjects with PA after treatment. Data are expressed as the mean ± SD; *P* < 0.001, paired *t* test for changes from baseline. **C** Changes in pendrin levels from baseline in subjects with PA who underwent adrenalectomy (Ope) or those treated with MR antagonists without surgical intervention (Med). *P < 0.05, **P < 0.01, paired *t* test for changes from the baseline. Data are expressed as the mean ± SD.

We examined whether the pendrin levels in urinary EVs were altered by treatment in the patients with PA. The pendrin levels in urinary EVs were analyzed by Western blots before and after treatment (unilateral adrenalectomy and pharmacological MR blockade) in 13 patients. The baseline characteristics and treatment for each of the patients are shown in Supplementary Table 2. As shown in Fig. 4A and B, there was a significant reduction in pendrin abundance from baseline in the subjects with PA after treatment (49% reduction; *P* < 0.001). A previous study showed that MR antagonists and adrenalectomy are equally effective in patients with PA [44]. Consistently, we found no significant difference in the magnitude of the effects on pendrin reduction between those who received adrenalectomy and those who were treated with MR antagonists without surgical intervention. Both groups showed a significant reduction in pendrin levels compared with the baseline levels, although the effects seemed more variable in the latter group (Fig. 4C).

**Fig. 5:**
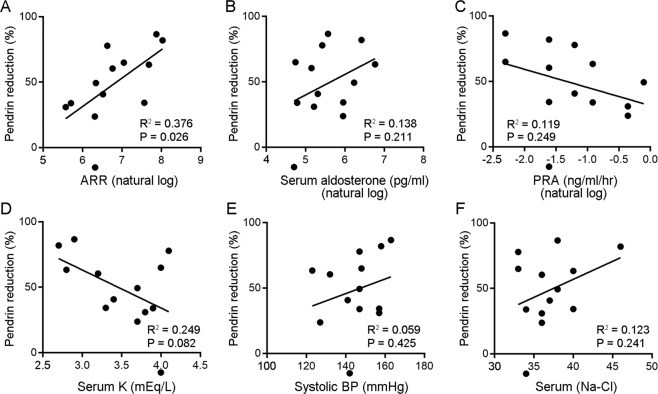
Scatter plot showing the relationship between changes in pendrin levels after treatment and baseline aldosterone-renin ratio (ARR) (**A**), serum aldosterone (**B**), plasma renin activity (PRA) (**C**), serum K^+^ (**D**), systolic blood pressure (BP) (**E**), and serum [Na^+^]-[Cl^−^] differences (a surrogate for serum bicarbonate levels in subjects with PA; see text) (**F**).

To obtain insights into the factors associated with the reduction in pendrin abundance, we performed correlation analysis using several clinical parameters obtained at baseline. We found that the magnitude of pendrin reduction after treatment was significantly correlated with the log-transformed aldosterone-renin ratio (ARR) at baseline (*R*^2^ = 0.376; *P* = 0.026) (Fig. 5A). As shown in Fig. 5B and C, no significant correlation was found for serum aldosterone levels or plasma renin activity (PRA). The lack of a significant association with serum aldosterone levels may be related to its high variability [45] and to the limited number of tested samples in the current study.

Existing evidence indicates the key role of pendrin in fluid volume homeostasis, potassium balance, and acid/base regulation [7, 17, 22, 46]. We next analyzed the association of pendrin levels with serum K^+^ and BP levels. The pendrin levels tended to be inversely correlated with the baseline serum K^+^ levels (*R*^2^ = 0.249; *P* = 0.082; Fig. 5D). However, we found no significant correlation between pendrin reduction and systolic BP (Fig. 5E), which can be explained by the fact that most of the patients received antihypertensives to control their BP. Because serum bicarbonate levels were not available in most of the study participants, we used the serum [Na^+^]-[Cl^−^] difference as a surrogate estimate of serum bicarbonate levels in PA (the value correlated well with serum bicarbonate levels in our rat PA model; Supplementary Fig. 1). As shown in Fig. 5F, there was no significant correlation between pendrin reduction and the serum [Na^+^]-[Cl^−^] difference.

### Correlation of pendrin in urinary EVs with ARR in patients with PA

**Fig. 6:**
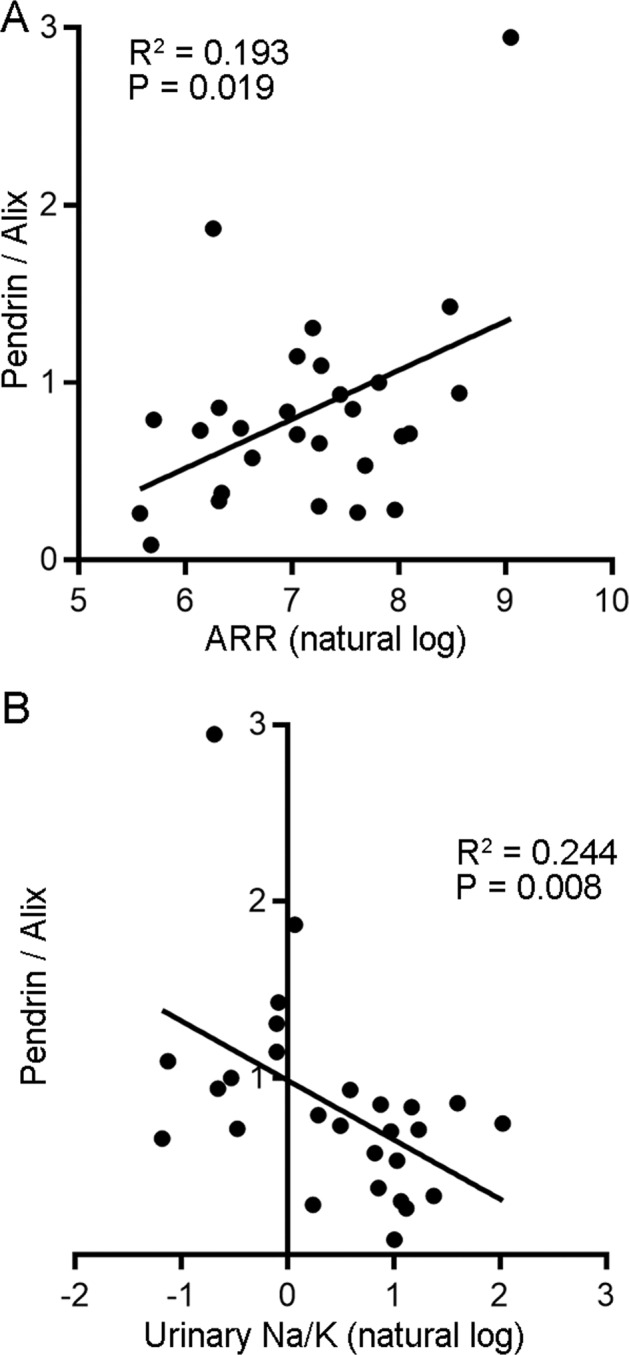
Correlation analysis of pendrin abundance in urinary EVs with ARR (**A**) and urinary Na^+^/K^+^ (**B**) among subjects with PA who received neither adrenalectomy nor MR antagonists.

To validate the correlation between pendrin and ARR, we finally evaluated the association between pendrin abundance in urinary EVs and ARR in 28 patients with PA who did not receive MR antagonists or adrenalectomy at the time of sample collection. The clinical characteristics are shown in Supplementary Table 3. Correlation analysis revealed that pendrin abundance corrected for Alix was significantly correlated with ARR (*R*^2^ = 0.193; *P* = 0.019) (Fig. 6A), confirming the relationship observed in the aforementioned study. In addition, similar to a previous study that analyzed ENaC in detail in urinary EVs from hypertensive subjects [33], we found that pendrin abundance in urinary EVs was significantly correlated with the urinary Na^+^/K^+^ ratio in the patients with PA (R^2^ = 0.244; *P* = 0.008).

## Discussion

We demonstrated that pendrin is detected in dimeric and monomeric forms in urinary EVs, which correlates with renal pendrin abundance in a rat model of aldosterone excess. We also found that the pendrin levels in urinary EVs are significantly attenuated by adrenalectomy or by pharmacological MR blockade in patients with PA; the magnitudes of changes are associated with ARR before treatment. Finally, cross-sectional analysis revealed that the pendrin levels in urinary EVs are significantly correlated with ARR. These data are consistent with experimental studies showing the stimulation of pendrin in a state of aldosterone excess, suggesting its role in aldosterone-induced fluid and electrolyte abnormalities [7, 10, 11, 17].

In our study, pendrin was detected as a monomer as well as a dimer in urinary EVs. Both signals in urinary EVs were eliminated by competition with the immunizing pendrin peptide, confirming the signal specificity. Pendrin was detected mainly as a monomeric form in the kidney lysates (Fig. 2C, left lane), and these differences may be attributable to experimental conditions. Biologically, SLC26 family members, including pendrin, can form dimers within the lipid bilayer through the interaction of the C-terminal domain [47, 48]. Notably, two cycles of pendrin in association with Na^+^-driven Cl^-^ and bicarbonate exchanger (NDCBE) have been proposed to mediate electroneutral NaCl transport in the collecting duct [49], which might be relevant for the dimeric organization observed in this study.

We infer that the changes in pendrin abundance affect fluid volume status in PA patients. Experimental studies have shown that pendrin is stimulated in response to the activation of the RAA system [16, 21–23] and that pendrin knockout mice show lower BP than wild-type mice under NaCl restriction [9, 10], demonstrating that the salt-retaining ability of the RAA system is in part mediated by pendrin. In addition to Cl^−^ reabsorption in exchange for HCO_3_^−^ secretion, the pendrin-ENaC interaction through luminal HCO_3_^−^ and ATP regulates NaCl retention [7]. Upregulation of pendrin expression also contributes to BP elevation in aldosterone excess because hypertension induced by mineralocorticoids was not observed in pendrin knockout mice [11], whereas these mice showed worsening of metabolic alkalosis and hypokalemia [11, 17]. The mechanisms regulating pendrin are multifactorial, and both MR-dependent and MR-independent pathways contribute to pendrin stimulation [14, 18, 22, 50]. In humans, Qi et al. reported that pendrin peptides in urinary EVs are increased by salt restriction in essential hypertension [33]. More recently, another proteomic study showed a decrease in pendrin peptides in urinary EVs in response to K^+^ supplementation during fludrocortisone loading, as well as the association between pendrin and aldosterone in patients with PA [19]. In our study, we demonstrated that pendrin in urinary EVs is significantly decreased by MR blockade or adrenalectomy in patients with PA. Overall, these data indicate that pendrin is altered in human hypertension and that the pendrin abundance in urinary EVs merits further investigation. In addition, given that the activity of renal NaCl transport mechanisms is a major determinant of salt sensitivity (BP response to salt intake) [51, 52], it will be of interest to analyze the relationship between salt sensitivity and the abundance of pendrin and other membrane proteins in urinary EVs in future studies.

Our study has several limitations. This study included a relatively small number of patients with PA because we isolated and analyzed proteins in urinary EVs in each of the patients with PA by Western blot analysis. The development of a high output approach such as ELISAs would overcome this barrier. In the cross-sectional analysis, a subpopulation of patients received renin-angiotensin system inhibitors or β blockers, which may have affected ARR in these patients; a confirmatory study with a larger number of patients with PA would be desirable and can also include other proteins such as NCC and ENaC. Another limitation is that we did not include healthy control subjects.

Despite these limitations, our study provides detailed biochemical characterization of pendrin in urinary EVs in patients with PA and demonstrates that it can be controlled by therapeutic interventions. Using a rat model, we also showed that the abundance of pendrin in urinary EVs, as well as that of ENaC and NCC, correlates with renal levels. Detailed analysis of urinary EVs in future studies may lead to the identification of useful biomarkers that can help guide optimal treatment of PA and other forms of human hypertension.

## Supplementary information

Supplementary Material
