# Supplementary material for: Characterization of pendrin in urinary extracellular vesicles in a rat model of aldosterone excess and in human primary aldosteronism
Source: Hypertens Res. 2021 Jul 29;44(12):1557–67. doi: 10.1038/s41440-021-00710-5 (PMC8645477; doi:10.1038/s41440-021-00710-5)

Supplemental Information for:

Characterization of pendrin in urinary extracellular vesicles in rat model of aldosterone excess and in human primary aldosteronism

Fumika Ochiai-Homma et al.

Supplementary Table 1. Biological parameters in aldosterone-infused rats.

Group	Control	Aldo	Aldo+Esax
Body weight (g)	462±33	485±46	459±47
Kidney weight (mg)	1.52±0.05	3.25±0.37[a]	2.42±0.22[c]
Systolic BP (mmHg)	123±5	150±11[b]	133±14
HR (bpm)	374±34	358±39	400±59
Serum Na (mmol/l)	134.4±5.9	139.0±0.8	138.8±0.5
Serum K (mmol/l)	4.3±0.5	2.3±0.2[a]	3.7±0.7[c]
Serum Cl (mmol/l)	100.3±1.0	86.8±1.5[a]	96.7±1.5[c]
Serum HCO_3 (mmol/l)	27.5±4.6	45.9±2.0[a]	33.6±2.5[c]
Urinary volume (ml)	23.9±8.3	44.0±13.6[a]	37.5±9.9
Urinary Na (mmol/day)	2.00±0.91	2.15±0.68	1.35±0.53
Urinary K (mmol/day)	3.27±1.07	4.31±0.75	4.15±1.34
Urinary Cl (mmol/day)	2.51±1.39	3.07±0.60	2.20±1.08
FEK (%)	18.0±6.1	54.2±8.4[a]	32.9±13.9[c]
Urinary albumin (mg/day)	0.13±0.13	10.53±3.56[a]	0.90±0.97[c]

Data are expressed as mean ± SD; [a]P < 0.01 versus Control; [b]P < 0.05 versus Control; [c]P < 0.01 versus Aldo.

Supplementary Table 2. Baseline characteristics of PA subjects in pre- and pos-treatment analysis.

Sex	PA subtype	Age at diagnosis	BMI	sBP/dBP	Serum Na	Serum K	Serum Cl	Serum (Na-Cl)	Urinary Na	Urinary K	Urinary Cl	DM	eGFR	PRA	Serum aldosterone	ARR	treatment	sBP/dBP (post-treatment)	Serum Na (post-treatment)	Serum K (post-treatment)	Serum Cl (post-treatment)	Urinary Na (post-treatment)	Urinary K (post-treatment)	Urinary Cl (post-treatment)
M	APA	47	33.0	157/98	145	3.3	105	40	101	42.0	94	(-)	59	0.2	387	1935	operation	130/85	142	4.4	107	65	43.0	62
M	IHA	47	26.8	141/89	142	3.4	105	37	149	19.6	123	(-)	73	0.3	203	677	medication	122/71	145	4.1	108	83	16.7	65
F	IHA	55	24.7	127/83	142	3.7	106	36	83	16.7	65	(-)	70	0.7	386	551	medication	118/78	141	4.4	104	49	30.6	58
F	IHA	47	20.2	147/68	141	4.1	108	33	35	15.4	34	(-)	97	0.3	227	757	medication	133/77	141	4.1	108	195	67.1	131
M	APA	40	26.0	158/98	145	2.7	99	46	54	20.4	47	(-)	53	0.2	616	3080	operation	118/72	140	4.7	103	63	28.2	71
M	IHA	45	28.7	148/105	141	4.0	108	33	43	47.6	66	(-)	95	0.1	115	1150	medication	144/96	142	4.2	108	173	58.6	128
M	IHA	56	22.5	147/90	143	3.9	109	34	88	66.0	115	(-)	73	0.4	120	300	medication	120/68	144	3.7	107	169	56.6	185
F	IHA	51	19.8	142/79	142	4.0	108	34	42	10.6	44	(-)	81	0.2	111	553	medication	117/75	138	4.1	104	43	17.1	43
M	IHA	56	27.6	157/104	142	3.8	106	36	110	35.9	90	(-)	70	0.7	184	263	medication	120/85	139	5.0	102	112	59.4	125
F	APA	46	19.5	132/81	140	3.2	104	36	64	19.8	79	(-)	94	0.2	173	865	operation	124/84	138	4.1	103	154	46.5	132
M	APA	61	27.6	147/88	145	3.7	107	38	72	30.6	79	(-)	69	0.9	510	567	operation	129/88	145	3.6	110	157	13.3	152
F	APA	65	21.1	163/92	147	2.9	109	38	55	8.6	55	(-)	80	0.1	263	2630	operation	112/72	141	4.1	105	66	41.4	90
M	APA	42	22.7	123/80	146	2.8	106	40	89	31.7	110	(-)	81	0.4	871	2178	operation	140/80	139	4.7	103	56	9.5	57

Supplementary Table 3. Characteristics of patients participated in the cross-sectional analysis.

Age, years	50.1 (11.9)
Mele	18 (64%)
Female	10 (36%)
Body mass index, kg/m^2	25.2 (4.2)
SBP, mmHg	143.1 (19.1)
DBP, mmHg	87.2 (15.3)
Serum K^+, mEq/L	3.29 (0.47)
Serum Na^+, mEq/L	143.5 (2.2)
Serum Cl^-, mEq/L	105.6 (2.8)
eGFR, ml/min/1.73m^2	79.1 (17.5)
Diabetes mellitus	2 (7%)
PA subtype	
Unilateral adrenal lesion/aldosterone-producing adenoma	15 (54%)
Bilateral adrenal lesion/idiopathic hyperaldosteronism	11 (39%)
Unknown	2 (7%)
PAC, pg/mL	423.4 (276.9)
PRA, ng/ml/h	0.33 (0.22)
ARR	1862 (1843)
Antihypertensive treatment	27 (96%)
CCB	25 (89%)
ACEI/ARB	4 (14%)
α–blocker	6 (21%)
β–blocker	2 (7%)
K^+ supplement	17 (61%)

Data are n (%) or mean (SD).

Supplementary Figure 1. Serum [Na^+]–[Cl^-] difference highly correlates with serum HCO_3 levels in the rat model. Correlation between serum [Na^+]–[Cl^-] difference and HCO_3 levels was analyzed across control rats, aldosterone-infused rats, and aldosterone-infused rats receiving esaxerenone (R^2 = 0.978; $P < 0.0001$).

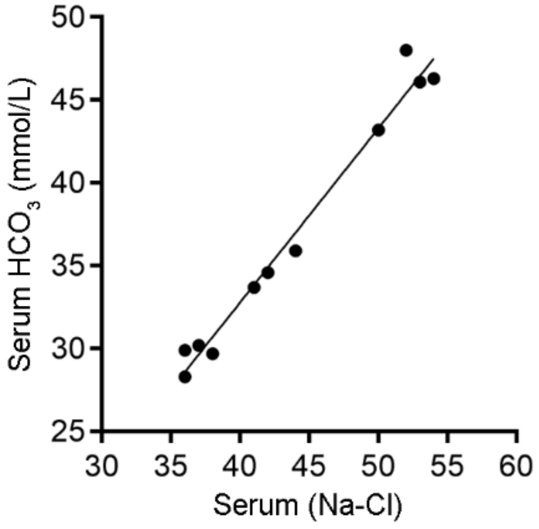

Supplement: Supplementary file 1 — Supplementary Material [file 41440_2021_710_MOESM1_ESM.pdf]
